# Heterogeneity of uroplakin localization in human normal urothelium, papilloma and papillary carcinoma

**DOI:** 10.2478/raon-2013-0052

**Published:** 2013-10-08

**Authors:** Dasa Zupancic, Rok Romih

**Affiliations:** Institute of Cell Biology, Faculty of Medicine, Ljubljana, Slovenia

**Keywords:** human urothelium, papilloma, papillary carcinoma, uroplakins, immunoelectron microscopy, immunohistochemistry

## Abstract

**Background:**

Uroplakins are differentiation-related membrane proteins of urothelium. We compared uroplakin expression and ultrastructural localization in human normal urothelium, papilloma and papillary carcinoma. Because of high recurrence rate of these tumours, treated by transurethral resection, we investigated urothelial tumour, resection border and uninvolved urothelium.

**Patients and methods:**

Urinary bladder samples were obtained from tumour free control subjects and patients with papilloma and papillary carcinoma. Immunohistochemical and immunoelectron labelling of uroplakins were performed.

**Results:**

In normal human urothelium with continuous uroplakin-positive superficial cell layer uroplakins were localized to flattened mature fusiform vesicles and apical plasma membrane of umbrella cells. Diverse uroplakin expression was found in papilloma and papillary carcinoma. Three aberrant differentiation stages of urothelial cells, not found in normal urothelium, were recognized in tumours. Diverse uroplakin expression and aberrant differentiation were occasionally found in resection border and in uninvolved urothelium.

**Conclusions:**

We demonstrated here that uroplakin expression and localization in urothelial tumours is altered when compared to normal urothelium. In patients with papilloma and papillary carcinoma immunolabelling of uroplakins at ultrastructural level shows aberrant urothelial differentiation. It is possible that aberrant differentiation stages of urothelial cells in resection border and in uninvolved urothelium contribute to high recurrence rate.

## Introduction

Urothelium is the source of most urinary bladder cancers, which are the ninth most common malignancy worldwide.^1^ Among all bladder urothelial cancers, more than half represent the papilloma and the papillary carcinoma.^2^ Tumours, which are cytopathologically and recently even cytogenetically diagnosed by the examination of urine or bladder washing^3^, are usually resected during the transurethral resection of the urinary bladder (TURB).^4^ However the recurrence rate of this malignancies is nearly 70%.^5^

Four uroplakins (UPIa, UPIb, UPII and UPIIIa)^6^,^7^ are the major differentiation products of urothelium^8^,^9^, however their expression is not strictly correlated with pathological stage and grade.^10^–^14^ Uroplakins are transmembrane proteins, which form complex membrane structures, called urothelial plaques^15^,^16^, which represent the ultimate marker for terminal differentiation of superficial umbrella cells of normal urothelium. Urothelial plaques cover up to 90% of the apical plasma membrane and fusiform vesicles.^15^ Fusiform vesicle undergoes distinct stages of maturation, from small, rounded uroplakin-positive transporting vesicles to discoidal immature fusiform vesicles and flattened mature fusiform vesicles, which are incorporated into the apical plasma membrane.^17^,^18^ In partially differentiated intermediate urothelial cells only uroplakin-positive transporting vesicles and immature fusiform vesicles are formed.^17^,^18^ Ultrastructural localisation of uroplakins is therefore closely related to the differentiation stage of urothelial cell. We have shown recently that ultrastructural localization of uroplakins is preserved in preneoplastic urothelium^19^, but the ultrastructural localization of uroplakins in urothelial tumour cells is not known.

The aim of the present study was to explore uroplakin expression and ultrastructural localization of uroplakins in normal urothelial cells and in urothelial cells of papilloma and papillary carcinoma from patients which underwent TURB. We analysed the tumour, the urothelium positioned next to the resected tumour, which we termed “re-section border”, and the urothelium away from the removed tumour, which we termed “uninvolved urothelium”. We showed that ultrastuctural localization of differentiation-related uroplakins may be featuring different urothelial regions in patients with urothelial tumours.

## Materials and methods

### Patients and sampling

The study was approved by the Slovenian National Medical Ethics Committee, No. 80/11/99, and was conducted in accordance with the Helsinki Declaration. The study population consisted of 25 patients with bladder cancer who underwent TURB and 4 tumour free control subjects who underwent transurethral resection of the prostate. Patients were histopathologically diagnosed as papilloma (5 patients; 41 to 76 y-old; mean age 55,6 y), papillary urothelial carcinoma - pTa (15 patients; 9 patients with G1, 3 patients with G1–2, 2 patients with G2 and 1 patient with G2–3; 46 to 77 y-old; mean age 65,8 y), and papillary urothelial carcinoma with lamina propria invasion - pT1 (5 patients; 1 patient with G1–2, 3 patients with G2 and 1 patient with G2–3; 64 to 73 y-old; mean age 67,3 y). The 1998 “World Health Organization/International Society of Urological Pathology consensus classification of urothelial (transitional cell) neoplasms of the urinary bladder” was used for pathologic staging and grading.^20^ All control subjects were diagnosed as benign prostatic hyperplasia.

From each control and papilloma one sample was obtained. From papillary urothelial carcinoma (pTa, pT1) three samples were acquired: (i) the urothelial tumour, (ii) the urothelium positioned next to the resected tumour, *i.e*. “resection border”, and (iii) the urothelium away from the removed tumour, *i.e*. “uninvolved urothelium”. Each sample was cut into 2 halves immediately after biopsy, one being processed for light and the other for the electron microscopy.

### Immunohistochemical labelling of uroplakins

For light microscopy, samples were immersed in Bouin solution for 24 h, dehydrated and embedded in paraffin wax. Paraffin sections were cut from at least two different parts of each sample. Sections were incubated in the anti-uroplakins antibody.^6^ Sections were then labelled with biotinylated swine anti-rabbit immunoglobulins (Dako, Glostrup, Denmark), followed by ABC/HRP complex (Vector Laboratories, Burlingame, CA), developed by DAB (Sigma, Taufkirchen, Germany) and counterstained with haematoxylin. For negative controls, the incubation with primary antibody was omitted or the specific primary antibody was replaced by a non-relevant antibody. Sections were examined with a Nikon Eclipse TE300 microscope.

### Immunoelectron microscopy

For immunoelectron microscopy, samples were cut into 1mm^3^ pieces and fixed in 2% paraformaldehyde plus 0.05% glutaraldehyde. Samples were dehydrated by increasing the concentration of ethanol while simultaneously decreasing the temperature and then they were embedded in Lowicryl HM20 resin at −50°C. The resin was polymerized by ultraviolet light. Semithin and ultrathin sections were cut. Semithin sections were immunolabelled first in order to find appropriate region for immunoelectron microscopy. Both sections were treated according to the same protocol. Non-specific labelling was blocked by the PBS buffer containing 0.1% fish gelatin, 0.8% bovine serum albumin and 5% fetal calf serum (blocking buffer). Sections were incubated with anti-uroplakins antibody in the blocking buffer. After washing in washing buffer (blocking buffer without fetal calf serum) primary antibody was detected with goat anti-rabbit IgG conjugated to either 5 nm or 10 nm colloidal gold, diluted 1:50 in blocking buffer. Sections were washed in washing buffer followed by ultrapure water. The 5 nm gold was silver enhanced with IntenSE (Amersham, U.K.). The enhancement times were 24 min and 4 min for semithin and ultrathin sections, respectively. Semithin sections were counterstained with toluidin blue and examined under an epi-polarization microscope in transmitted bright-field (Axioscop 20, Carl Zeiss). Ultrathin sections were counterstained with uranyl acetate and lead citrate and viewed in a Philips CM100 transmission electron microscope.

## Results

Different immunohistochemical labelling of uroplakins is displayed in human normal urothelium, papilloma and papillary carcinoma

According to WHO consensus classification^20^, control subjects with benign prostatic hyperplasia had a normal urothelium, with no cytological atypia, but with slight hyperplasia in some areas, which was in agreement with previous studies.^12^,^21^ Several areas of urothelium showed normal morphology with three to five cell layers and large superficial umbrella cells, while other areas of urothelium consisted of up to seven cell layers with smaller superficial cells. No other premalignant or malignant changes were observed.

**FIGURE 1.**
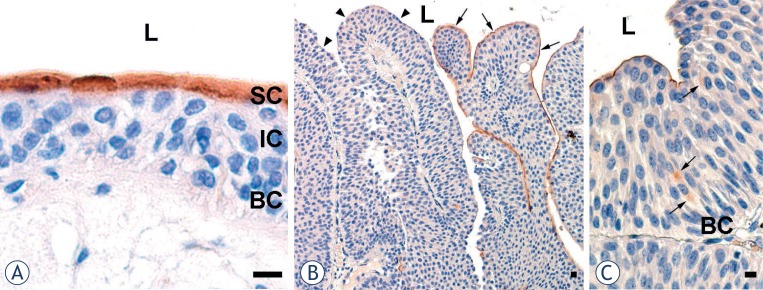
Immunohistochemical labelling of uroplakins in normal urothelium and in papiloma (**A**) Brown reaction products shows that superficial cells (SC) with high uroplakin expression form a continuous layer in normal urothelium from control subjects. Underlying intermediate (IC) and basal cells (BC) are uroplakin-negative. (**B**) In the urothelium of a patient with papilloma, superficial cells of some papilae are uroplakin-positive (arrows), while others are uroplakin-negative (arrowhead). (**C**) Individual intermediate urothelial cells of papilae are uroplakin-positive (arrows). L – lumen. Scale bars: 10 μm.

Consistent with the notion that uroplakins are expressed in terminally differentiated umbrella cells^8^,^9^, we considered as normal urothelium only the regions where immunohistochemical labelling of uroplakins was positive in all superficial cells (Figure 1A).

Papilloma was defined as a discrete papillary growth with a central fibrovascular core lined by urothelium of normal thickness and cytology.^20^ The apical cell layer of papillae was composed of uroplakin-positive and uroplakin-negative regions (Figure 1B). Although all basal cells and the majority of intermediate cells of papillae were uroplakin-negative, rare intermediate cells exhibited positive uroplakin labelling (Figure 1C).

**FIGURE 2.**
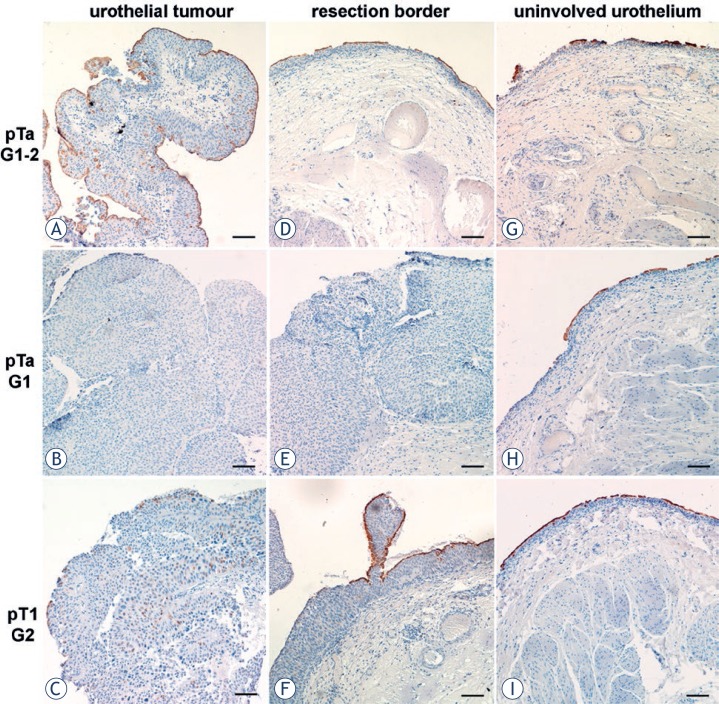
Immunohistochemical labelling of uroplakins (brown reaction products) in urothelial tumour (**A–C**), resection border (**D–F**) and uninvolved urothelium (**G–I**) from patients with noninvasive (pTa) or lamina propria invasive (pT1) papillary carcinomas. Urothelial tumour with (**A**) uroplakin-positive superficial cell layer and individual uroplakin-positive intermediate cells, (**B**) uroplakin-negative urothelium and (**C**) urothelium containing rare uroplakin-positive cells. Resection border with (**D,F**) uroplakin-positive and uroplakin-negative regions of urothelium and (**E**) completely uroplakin-negative urothelium. Uninvolved urothelium with (**G,H**) small and (**I**) large regions of uroplakin-positive superficial cells. In the region with uroplakin-positive superficial cells, some intermediate cells are also uroplakin-positive. Scale bars: 100 μm.

Papillary carcinomas showed no direct correlation between uroplakin expression and carcinoma staging and grading, which was consistent with previously published data.^12^,^13^ In order to compare uroplakin expression in different urothelial regions, immunohistochemical labelling of uroplakins was performed on the paraffin sections from (i) the urothelial tumour, (ii) resection border and (iii) un-involved urothelium. In general, uroplakin expression was lower in all patients compared to normal urothelium. (i) Urothelium of urothelial tumours showed three distinct types of uroplakin labelling patterns. In the first type, urothelium was covered by continuous layer of uroplakin-positive superficial cell and it also contained individual uroplakin-positive intermediate cells (Figure 2A). In the second type, urothelium was completely uroplakin-negative (Figure 2B). In the third type, urothelium contained individual uroplakin-positive cells, which were scattered throughout all cell layers (Figure 2C). (ii) Urothelium of resection borders also revealed heterogeneous labelling patterns, which could be placed into three categories. One type of resection border contained urothelium with normal histology, but discontinuous uroplakin expression in the superficial cell layer (Figure 2D). Another type of resection border was composed of urothelium, which had the same histopathologic characteristics as tumours. Here, urothelium was also completely uroplakin-negative as was the case in the neighbouring tumour (Figure 2E). Yet another type of resection border contained hyperplastic urothelium, which was covered with large stretches of uroplakin-positive superficial cells, alternating with large stretches of uroplakin-negative superficial cells. Urothelial regions covered with uroplakin-positive superficial cells contained uroplakin-positive intermediate cells. These uroplakin-positive cells were always found just beneath the superficial cell layer (Figure 2F). (iii) Uninvolved urothelia exhibited normal histology; however their superficial cells were either uroplakin-positive or uroplakin-negative (Figure 2G–I). The extent of uroplakin-positive versus uroplakin-negative regions varied to a great degree. As described for tumours and resection borders, uninvolved urothelium also contained individual uroplakin-positive intermediate cells, usually underlying uroplakin-positive superficial cells (Figure 2I).

Uroplakin ultrastructural localization in papilloma and papillary carcinoma is altered in comparison to normal human urothelium

Because immunohistochemical labelling of uroplakins is insufficient for determination of urothelial cell’s differentiation stage, we performed immunoelectron microscopy to determine ultrastructural localization of uroplakins in human normal and tumour urothelial samples.

**FIGURE 3.**
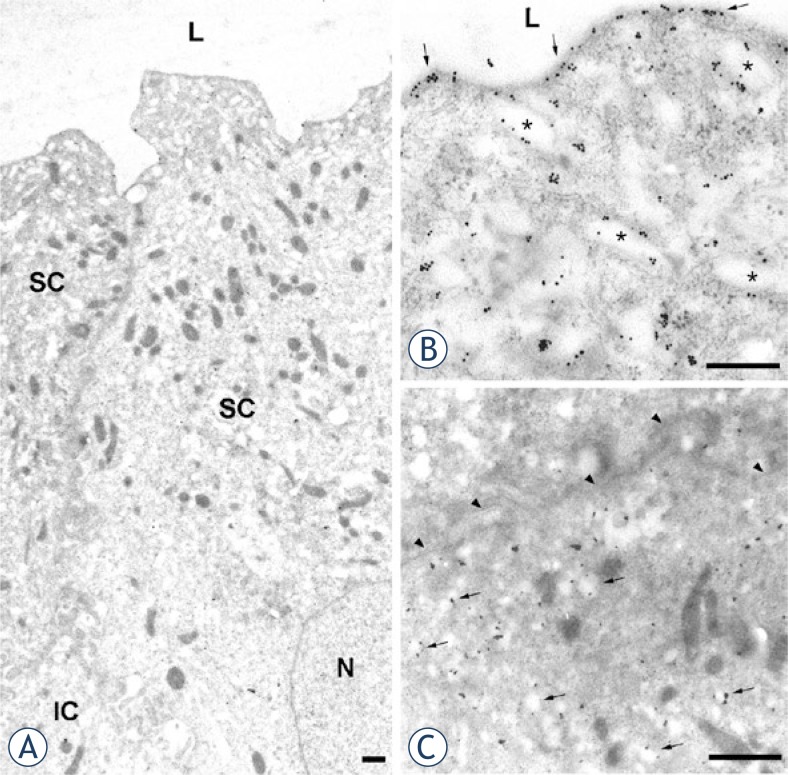
Immunoelectron microscopy of uroplakins in normal urothelium. (**A**) Two neighbouring superficial umbrella cells (SC), contain numerous mature fusiform vesicles. A part of underlying intermediate cell (IC) is also seen. (**B**) In umbrella cell uroplakin-positive apical plasma membrane (arrows) and uroplakin-positive mature fusiform vesicles (asterisks) are heavily labelled with colloidal gold particles. (**C**) Intermediate cell contains uroplakin-positive transporting vesicles (arrows). The basolateral plasma membrane (arrowheads) is uroplakin-negative. N – nucleus, L – lumen. Scale bars: 1 μm.

Normal urothelium was covered with superficial cells, which contained uroplakin-positive large flattened mature fusiform vesicles and uroplakin-positive apical plasma membrane (Figure 3A, B). These superficial cells were terminally differentiated and they shared characteristics with umbrella cells described in other species.^16^,^18^ Intermediate cells of normal urothelium were generally uroplakin-negative, except for some intermediate cells that contained small, round uroplakin-positive transporting vesicles. Their plasma membrane was always uroplakin-negative (Figure 3C), which is also in agreement with observations in other species.^18^

**FIGURE 4.**
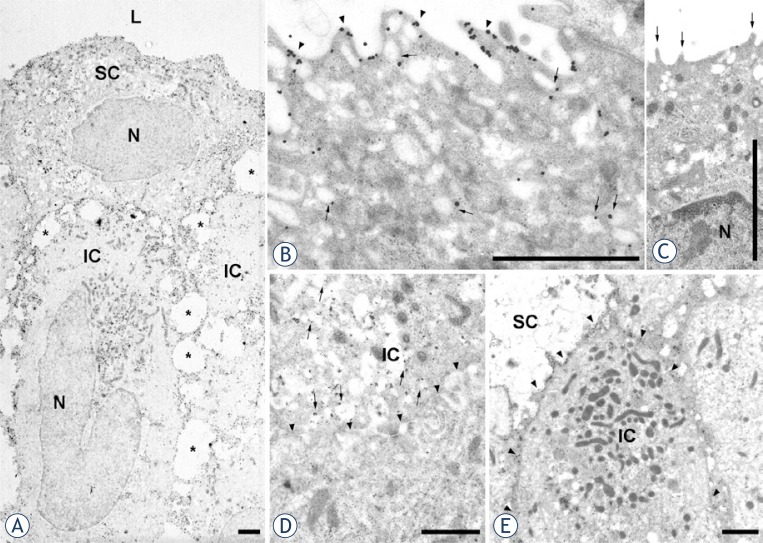
Immunoelectron microscopy of uroplakins in urothelial tumours. (**A,B**) papilloma, (**C**) tumour region of pTa G1, (**D**) tumour region of pT1 G1–2, (**E**) uninvolved urothelium of pTa G1–2. (**A**) Uroplakin-positive superficial (SC) and uroplakin-positive intermediate cells (IC) are shown. Note dilatations of intercellular spaces (asterisks) and prominent cytoplasmic processes that interconnect neighbouring cells. (**B**) Superficial cell with uroplakin-positive apical plasma membrane (arrowheads) and weakly positive immature fusiform vesicles (arrows). (**C**) Uroplakin-negative superficial cell with microvilli (arrows) on the apical surface. (**D**) Intermediate cell with uroplakin-positive transporting vesicles (arrows) and uroplakin-negative plasma membrane (arrowheads). (**E**) Intermediate cell with numerous mitochondria, uroplakin-positive transporting vesicles and uroplakin-positive plasma membrane (arrowheads). N – nucleus, L – lumen. Scale bars: 1 μm.

Immunoelectron microscopy of uroplakins confirmed the results of imunohistochemical labelling of urothelial tumours. We observed uroplakin-positive (Figure 4A, B) and uroplakin-negative (Figure 4C) superficial cells in papilloma and in all regions (tumour, resection border, uninvolved urothelium) of pTa and pT1 papillary carcinoma. Uroplakin-positive superficial cells had uroplakins in their apical plasma membranes and cytoplasmic vesicles. These uroplakin-positive vesicles were smaller and weakly labelled (Figure 4B) when compared to mature fusiform vesicles of normal urothelium (Figure 3B), and therefore represented immature fusiform vesicles. Importantly, mature fusiform vesicles were not detected in any of the samples taken from patients with urothelial tumours, not even in uninvolved urothelium. Uroplakin-negative superficial cells had microvilli on their apical surface and small, rounded vesicles in their cytoplasm (Figure 4C).

Also in agreement with imunohistochemical labelling, we observed uroplakin-positive intermediate cells in urothelial tumours (Figure 4A,D,E). One type of uroplakin-positive intermediate cells had uroplakin-positive transporting vesicles and uroplakin-negative plasma membranes (Figure 4D). This type of intermediate cells therefore had all the characteristics of corresponding intermediate cells of normal urothelium (Figure 3C). There was another type of uroplakin-positive intermediate cells that had never been described before. This type of cells was characterized by the presence of numerous mitochondria, uroplakin-positive transporting vesicles and uroplakin-positive plasma membranes (Figure 4E). However, the majority of intermediate (as well as all basal cells) were uroplakin-negative. All the described types of intermediate cells could be found in papilloma and in all samples from patients with papillary carcinoma.

## Discussion

The majority of knowledge about normal urothelium originates from the studies on laboratory animals. In the normal human urothelium, urothelial plaques and fusiform vesicles has not yet been extensively studied, partially due to the fact that urothelium is obtained from patients and not from healthy individuals. The definition from WHO/ISUP consensus^20^ proposes that “Flat lesions with benign cytology and minimal disorder should not be designated as mild dysplasia but rather as normal urothelium”. In our study we adopted more stringent criteria for normal urothelium, which must contain undisrupted layer of superficial umbrella cells expressing uroplakins. Umbrella cells have uroplakins in their apical plasma membrane and contain mature fusiform vesicles (Table 1), which are flat and strongly uroplakin-positive vesicles. Minority of urothelial regions fulfil this demand. This is probably because urothelia are from control subjects with benign prostatic hyperplasia, which decrease the differentiation stage of superficial urothelial cells in some areas.^12^ Intermediate urothelial cells have uroplakin-positive transporting vesicles, but their plasma membranes are uroplakin-negative. This is consistent with the normal mouse urothelium, where uroplakin-positive transporting vesicles are found, but plasma membrane of partially differentiated intermediate cells does not contain uroplakins.^17^ We assume that at least some intermediate cells synthesize uroplakins, which accumulate in uroplakin-positive transporting vesicles (Table 1). It seems that these vesicles cannot fuse with the plasma membrane.

Since identification of uroplakins as the major differentiation products of normal urothelium^8^,^9^ their altered expression in urothelial carcinomas has been studied. We demonstrate here, that in papilloma only some regions of apical surface and extremely rare intermediate cells express uroplakins. On the other hand, papillary carcinoma elaborates heterogeneous uroplakin distribution. Some tumours are almost entirely covered with superficial cells expressing uroplakins and they also contain individual intermediate cells expressing uroplakins. Other tumours express no uroplakins, while several of them exhibit numerous intermediate cells and rare superficial cells expressing uroplakins. Uroplakins are located in the apical plasma membrane as in normal urothelium, while no mature fusiform vesicles are detected in the cytoplasm of these superficial urothelial cells. The cytoplasm of these superficial urothelial cells contains immature fusiform vesicles (Table 1), similar to partially differentiated intermediate cells of normal mouse urothelium.^18^ Therefore, we assume that uroplakin expressing superficial urothelial cells of papilloma and papillary carcinoma take alternative pathway of differentiation and elaborate special aberrant differentiation stage. This might be interpreted as compensatory effect of upregulating major differentiation products to counter the proliferative effects of the tumour cells.^13^ Urothelial plaques may retain smaller as observed in preneoplastic mouse urothelium.^19^ In the majority of intermediate cells of papilloma uroplakins are absent, indicating their low differentiation stage. Rare intermediate cells, which express uroplakins, exhibit them in uroplakin-positive transporting vesicles and also in their plasma membrane (Table 1). These cells represent another unique aberrant differentiation stage. Similarly, in another type of intermediate cells of papillary carcinoma, uroplakins are localized in uroplakin-positive transporting vesicles and not in the plasma membrane. To sum up, cells of urothelial tumours, which express uroplakins, could follow diverse aberrant differentiation pathways as observed by uroplakin localization analysis. This is probably due to the fact that uroplakin expression and transport is subject to different regulatory mechanisms, which need to be further exploited.

Urothelial tumours have high recurrence rates^5^, however the exact reasons for that are not yet known. Our study revealed that uninvolved urothelia and resection borders have disrupted uroplakin-positive superficial cell layer and therefore does not fulfil all the criteria for normal urothelium. Since resection borders are usually not removed during TURB, even more pronounced concern represents our finding, that the majority of them were composed of hyperplastic urothelium or even urothelium, which had architectural and cytological characteristics and uroplakin expression similar to that in the tumour. Therefore it is possible that although the main portion of urothelial tumour is removed during TURB, potentially dangerous parts of urothelium are often left behind.

In conclusion, when carefully selected regions of normal human urothelium are examined, umbrella cells develop uroplakin-positive apical plasma membrane and mature fusiform vesicles. Uroplakin expression in the urothelial tumour cells results in heterogeneity of uroplakin ultrastructural localization and contribute to diverse unique aberrant cell differentiation. We assume that such cells in resection border and uninvolved urothelium might represent potential source of new malignant growth and probably contribute to high recurrence rate of this kind of tumours.

## Figures and Tables

**TABLE 1.** Uroplakin-positive structures in the urothelial cells of human normal urothelium, papilloma and papillary carcinoma (urothelial tumour, resection border and uninvolved urothelium of pTa and pT1).

**Histology**	**Superficial cells**		**Intermediate cells**		**Basal cells**
normal urothelium	mFV, iFV, UPTV,	aPM	UPTV^*^		uroplakin
	
papilloma papillary carcinoma	iFV^*^,	aPM^*^	UPTV^*^,	PM^*^	negative

mFV = mature fusiform vesicles, iFV = immature fusiform vesicles, UPTV = uroplakin-positive transporting vesicles, aPM = apical plasma membrane, PM = plasma membrane

* individual cells (the majority of cells is uroplakin negative)
